# Tacrolimus as a Promising Drug for Epistaxis and Gastrointestinal Bleeding in HHT

**DOI:** 10.3390/jcm12237410

**Published:** 2023-11-29

**Authors:** Paloma Álvarez-Hernández, José Luis Patier, Sol Marcos, Vicente Gómez del Olmo, Laura Lorente-Herraiz, Lucía Recio-Poveda, Luisa María Botella, Adrián Viteri-Noël, Virginia Albiñana

**Affiliations:** 1 Otorrhinolaringology Department, Hospital Universitario Fundación Alcorcón, 28922 Madrid, Spain; palvarezhernandez@salud.madrid.org (P.Á.-H.);; 2 Internal Medicine Department, Hospital Universitario Ramón y Cajal, Instituto Ramón y Cajal Investigación Sanitaria, IRYCIS, 28034 Madrid, Spain; joseluis.patier@salud.madrid.org (J.L.P.); vicente.gomezol@salud.madrid.org (V.G.d.O.); 3 Faculty of Medicine and Health Sciences, Universidad de Alcalá (UAH), 28801 Alcalá de Henares, Spain; 4 CIBER Rare Diseases Unit 707, Centro de Investigaciones Biológicas Margarita Salas, Consejo Superior Investigaciones Científicas, CSIC, 28040 Madrid, Spain; laura.lorente@cib.csic.es (L.L.-H.); lucia.recio@cib.csic.es (L.R.-P.); cibluisa@cib.csic.es (L.M.B.)

**Keywords:** HHT, bleeding, epistaxis, GI bleeding, tacrolimus, ESS, hemoglobin

## Abstract

Background: Hereditary Hemorrhagic Telangiectasia (HHT) is a vascular autosomically inherited rare disease. Epistaxis (nose bleeds) is the most common symptom in HHT, leading to anemia and affecting the patient’s quality of life. In addition to epistaxis, gastrointestinal bleeding (GI), more often at older ages, may lead to severe anemia and the need for blood transfusions. Thus, finding drugs to control both types of bleeding is a primary necessity in HHT. Methods: A cross-sectional observational study was conducted in a series of 11 HHT patients treated with low tacrolimus doses (0.5–2 mg/day) on an off-label prescription basis. Patients showed refractory bleeding to previous treatments. The epistaxis severity score (ESS) and hemoglobin levels were the parameters used to evaluate the impact of tacrolimus. The occurrence of side effects was also recorded. Results: Tacrolimus was well tolerated in all of the patients except 2 (who stopped the treatment). The remaining patients tolerated the treatment, with a general improvement in their health condition. Epistaxis was significantly reduced when comparing the ESS before and after the treatment. Hemoglobin levels significantly increased, overcoming the anemia, during the course of the treatment. Conclusion: Tacrolimus at low doses should be considered as a promising treatment for epistaxis and gastrointestinal bleeding in HHT.

## 1. Introduction

Hereditary haemorrhagic telangiectasia (HHT) or Rendu–Osler–Weber syndrome is an autosomal dominant inherited vascular disease.

In 2000, the Curaçao criteria for clinical diagnosis of HHT were agreed upon. These criteria include multisystem symptoms such as spontaneous and recurrent epistaxis (nosebleeds), mucocutaneous telangiectasias, involvement of the visceral vasculature as gastrointestinal telangiectasis, arteriovenous malformations (AVMs) primarily affecting organs like the lung, liver, and brain, and having a first-degree relative with a confirmed diagnosis of HHT [1,2,3].

The prevalence of HHT is considered to be 1:5000 on average, although due to founder and isolation effects, in some locations including the Jura region (France), Funen Island (Denmark), and the Dutch Antilles, the prevalence is higher [3,4,5]. Heterozygous mutations in the *ACVRL1*/*ALK1* or *Endoglin* (*ENG*) genes are responsible for pathogenesis in about 85% of patients with HHT [6,7].

Less frequent HHT-causing mutations are detected in the *MADH4*/*SMAD4* gene, resulting in a joint condition known as Juvenile Polyposis and HHT (JPHT) [8], where, in addition to HHT symptoms, colon polyps and thoracic aneurysms appear [9]. In addition, Chromosomes 5 and 7 have been reported to contain two specific loci with genes of an unknown identity, responsible for causing HHT3 [10] and HHT4, respectively [11]. A syndrome similar to HHT, known as HHT5, is linked to mutations in *BMP9*/*GDF2* [12]. Importantly, all proteins encoded by these genes belong to the BMP9/TGF-β-signalling pathway.

Nosebleeds are the most frequent clinical symptom of HHT. Up to 93% of HHD patients suffer mild to moderate nosebleeds [13,14], which interferes with their quality of life [15]. Alongside epistaxis, gastrointestinal (GI) bleeding attributed to telangiectasias in the digestive tract is detected in as many as 80% of HHT patients, particularly in those of advanced age [16]. GI bleeding represents a major clinical problem, which may lead to a dependence on blood transfusions [17].

Typically, pharmaceutical therapies or minor surgical procedures treat the clinical symptoms of HHT.

Currently, there is no established optimal treatment for gastrointestinal bleeding. Nonetheless, certain systemic treatments designed for managing epistaxis may also offer potential benefits for addressing gastrointestinal bleeding. This paper will present a small case series using tacrolimus as a pharmacological treatment for very severe bleeding in HHT patients, including epistaxis and gastrointestinal bleeding.

**Figure 1:**
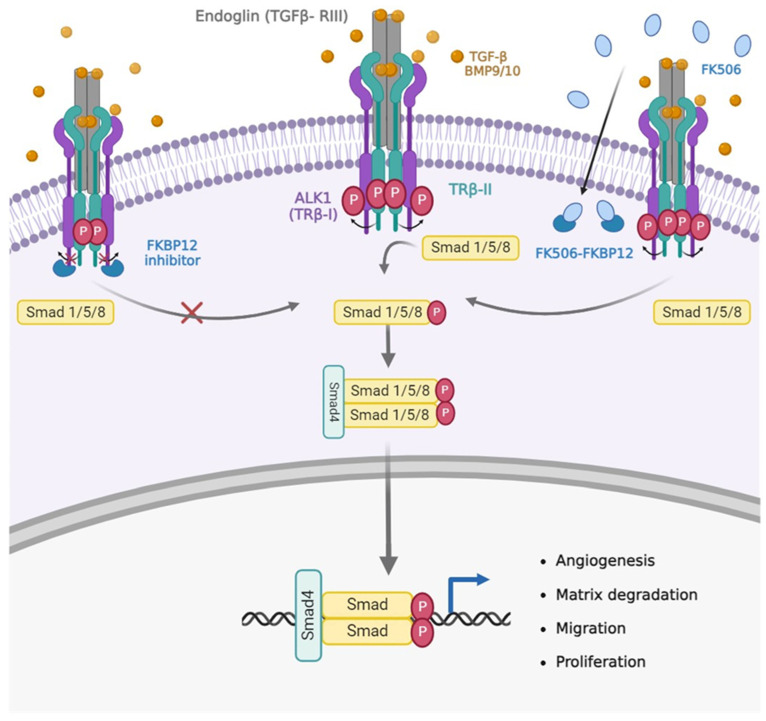
Hypothetic signalling pathway triggered by FK506. If the cell does not receive TGF-β/BMP9 angiogenic signals, the TRβ-I is inhibited by FKBP12, and the pathway is not initiated. In the presence of TGF-β/BMP9, the pathway will be activated with consequent phosphorylation of SMAD and expression of the target genes. After treatment with the immunosuppressant FK506, the same activation of the pathway is observed, as it sequesters the inhibitor of the TRβ-I, FKBP12, which can phosphorylate SMAD (modified from Albiñana et al., 2013 [20]. Created with BioRender.com, accessed on 2 October 2023).

The background for the interest in Tacrolimus or FK506 is historically linked to a case report involving a patient with HHT who underwent a liver transplantation [18]. To prevent rejection, the immunosuppressant FK506 was administered in combination with Aspirin and sirolimus to prevent rejection. One month after starting this treatment, the telangiectasias (both internal mucosa and external skin), epistaxis, and anemia were cured. This clinical case was the starting point for an in vitro study by Albiñana et al. [19,20] in an attempt to elucidate the molecular mechanism underlying this beneficial clinical effect on HHT. Endothelial cells were treated with tacrolimus alone, resulting in increased protein and mRNA expression of endoglin and ALK1. At the same time, stimulation of the BMP9/TGF-β1/ALK1-signalling pathway, as measured by the nuclear translocation of SMAD4 and the increased expression of downstream genes such as ID1, were obtained. These molecular findings were accompanied by enhanced endothelial cell functions such as tubulogenesis and wound healing [19,20] (Figure 1). These findings provide an explanation for the observed improvement in the patient’s condition resulting from a partial compensation of ALK1 and endoglin insufficiency, since the amount of both proteins and the signalling of the BMP9/TGF-β1/ALK1 pathway were stimulated.

In support of this view, Ruiz et al. (2017) documented that tacrolimus rescued gene expression dysregulations associated with ALK1 inhibition by increasing the ALK1 signalling pathway in endothelial cells derived from HHT patients. Furthermore, in an animal model of HHT immunotreated with antibodies against BMP9/10, tacrolimus improved vascular pathology by inhibiting VEGF signalling and thereby decreasing retinal hypervascularization [21].

Ruiz et al. more recently reported that in the same neonatal mouse model of HHT (BMP9/BMP10 immunosupressed mouse), the combination of sirolimus, an mTOR inhibitor, and nintedanib, an inhibitor of the tyrosine kinase receptor, exhibited a synergistic effect, completely inhibiting and even reversing retinal AVMs. In BMP9/10ib mice, the combination of sirolimus and nintedanib prevented vascular abnormalities in the oral mucosa, liver, and lungs, and it notably reduced gastrointestinal bleeding and anemia in adult mice with an inducible ALK1 deficiency [22].

Furthermore, findings from the TACRO clinical trial, which examined the effectiveness and safety of topically administered 0.1% tacrolimus nasal ointment, were made available in 2020 [23]. The results showed that, although there was no improvement six weeks after the treatment, the favorable tolerance and substantial decrease in the duration of epistaxis during the treatment phase encouraged the investigators to proceed with a phase III trial. This phase would involve a larger patient population and an extended treatment period, with the primary focus being the reduction in the duration of epistaxis during treatment.

The present study shows the results of 11 patients treated off-label with low doses of systemic tacrolimus, prescribed by HHT referral physicians in Spain. In all cases, patients suffered severe epistaxis and/or gastrointestinal bleeding refractory to other treatments. The results are not part of a clinical trial, but a collection of cases that may be useful to illustrate the efficacy and safety of systemic tacrolimus treatment.

## 2. Materials and Methods

### 2.1. Participants and Study Design

This study represents a cross-sectional investigation conducted starting in 2018. It involved Hereditary Hemorrhagic Telangiectasia (HHT) patients receiving care at two medical institutions: the Hospital Universitario Fundación Alcorcón (HUFA) and the HHT reference unit at the Hospital Universitario Ramón y Cajal. All the participants were adults, aged 18 years or older, with a confirmed diagnosis of HHT, which was based on clinical and/or genetic criteria. The criteria to offer tacrolimus treatment was either to have severe epistaxis/GI bleeding or to be blood transfusion dependent. In most cases, the patients had undergone other procedures without satisfactory results.

Tacrolimus was initiated after approval by the pharmacy committee as a compassionate use indication, and patients who had been on this treatment for at least 1 year were registered. The doses were modified according to tolerability, and no more than 2 mg/day were given to avoid its immunosuppressive effect (expert opinion). The main indication for initiation was ESS ≥ 4, despite local treatment and/or anemia secondary to recurrent epistaxis or gastrointestinal bleeding. In one case, P8, the treatment was started due to tongue and tonsil hemorrhages. All patients who were treated with tacrolimus were included in this study. These individuals were under the care of the Otorhinolaryngology Unit at HUFA and the Internal Medicine Unit at Ramón y Cajal Hospital.

In cases where patients experienced epistaxis (nosebleeds) or gastrointestinal (GI) bleeding that had been unresponsive to prior treatments, low doses of tacrolimus were recommended as a treatment option. It is important to note that the use of tacrolimus in these patients was considered off-label and administered on a compassionate basis. All the patients signed informed consent for this treatment, and the treatment plans for each patient received approval from the Pharmaceutical Committee and the Clinical Research Ethics Committee at HUFA and Ramón y Cajal Hospitals, respectively (approval code: 257/23).

### 2.2. Study Outcomes and Evaluations

The primary focus of the study was to assess the impact on the frequency and severity of nosebleeds, known as epistaxis, in individuals with Hereditary Hemorrhagic Telangiectasia (HHT). This assessment was conducted using the Hereditary Hemorrhagic Telangiectasia Epistaxis Severity Score (HHT-ESS). The HHT-ESS is an internationally recognized and standardized tool designed to objectively measure the severity of epistaxis in HHT patients [24]. The HHT-ESS score is derived from an analysis of six key parameters associated with epistaxis, which include the intensity, frequency, and duration of epistaxis, the presence or absence of anemia, the necessity of blood transfusions, and the requirement for medical intervention to manage the hemorrhage.

Additionally, the study involved monitoring hemoglobin levels at the beginning of the study and during follow-up assessments. The research also considered other factors such as changes in hemoglobin levels, any unintended side effects, and the rate of participant drop-outs.

### 2.3. Statistical Analysis

The mean ± SD represents the quantitative results. A Student’s *t*-test analysis was conducted to compare means. Statistical significance was considered when *p* was less than 0.05. The degree of significance is visually presented in figures as follows: (* *p* < 0.05; ** *p* < 0.01, *** *p* < 0.001).

## 3. Results

Table 1 shows the characteristics of 11 patients treated with oral tacrolimus as an off-label treatment. The sample has a median age of 55.8 [38.0] years and includes five women. All the patients presented epistaxis, and the other manifestations and complications were described as gastro-intestinal (GI) bleeding, pulmonary arteriovenous malformations (AVMs), and anemia. Patients were suffering from epistaxis, GI bleeding, or both, and the hemorrhages had been refractory to previous treatments, listed also in the same table. It is important to mention that the treatment with tacrolimus was combined with on-demand sclerotherapy in several cases. However, sclerotherapy became less frequent in those cases where tacrolimus was used. At least, four out of five patients treated on demand with sclerotherapy saw a reduced need for this procedure, more than 1 year since the beginning of tacrolimus treatment.

Of the 11 patients, there were five who had never undergone sclerotherapies. Of these five patients, P1, P2, P5, and P6 did not undergo sclerotherapies either before or after the initiation of tacrolimus treatment. In addition, P7 started on tacrolimus treatment after a previous nasal closure (Young’s procedure), so no sclerotherapy could be performed.

There was a different response for the remaining six patients who underwent sclerotherapies (P3, P4, and P8–11).

For patients P3, P4, and P10, they attended sclerotherapy every 3 months before tacrolimus treatment, on average. After the initiation of treatment, the need for sclerotherapy was spaced to 6–12 months. Thus, there was a reduction of at least 3 months in 50% of patients requiring sclerotherapy (3/6). In the case of P9, she was going for sclerotherapy every 3 months before treatment. After tacrolimus, she did not need sclerotherapy for 1 year and a half. Patients P8 and P11 were on sclerotherapy every month before tacrolimus. After treatment, they have so far not needed further sclerotherapy.

To conclude, following sclerotherapy, tacrolimus reduced the need for sclerotherapy in 50% of patients (P3, P4, and P10) in at least 3 months. In addition, no further sclerotherapy was needed in the other 50% of patients (P8, P9, and P11).

Patients were prescribed a low dose of tacrolimus, ranging from 0.5 to 2 mg/day, depending on the tolerance and the severity of the hemorrhages, as described in the Methods. The doses used were 2.5–5 times lower than doses commonly used in immunosuppressive therapy for solid organ transplantation. Tacrolimus shows reduced epistaxis when used for immunosuppression in HHT patients subjected to organ transplants [18].

In two cases, patients stopped treatment due to gastric intolerance, but aside from this side effect, their overall tolerance was quite good. In these cases patients should maybe start with lower doses and increase them more slowly. This opinion is drawn after looking at the good results in 80% of the patients.

One patient stopped treatment because he did not perceive any improvement 6 months after starting it. P2 and P5 stopped treatment for intolerance but had not been treated previously with sclerotherapy. In the case of P7, with Young’s procedure, no sclerotherapy could be performed. After stopping tacrolimus, P2 and P5 started with sclerotherapy, five procedures the first year. P5 started with sclerotherapy to control epistaxis, and he is doing well.

P2 and P5 discontinued treatment due to intolerance. Neither of these two patients had been treated prior to tacrolimus initiation with sclerotherapy. After their discontinuation of tacrolimus, P2 and P5 started sclerotherapy sessions. P5 started with sclerotherapies to control epistaxis. In the case of P7, on whom Young’s procedure was performed, no subsequent sclerotherapies were performed.

The evolution and treatment of patients who stopped tacrolimus were as follows: P2’s health condition worsened after stopping tacrolimus and starting first with sclerotherapy (five sclerotherapies per year). In 2022, he was receiving blood transfusions every month, and he recently started on bevacizumab. P5 started with sclerotherapies to control epistaxis, and he is doing well for the time being. P7’s health condition worsened after stopping tacrolimus, and he underwent two embolizations of maxilar and facial arteries. More recently, 3 months ago, he went through an embolization of a nasal arterio-venous malformation.

All the patients were following the Curaçao clinical criteria. Three patients did not have a genetic diagnosis. In one patient, no mutation was found in *ENG* or in *ACVRL1*/*ALK1.* Four patients were diagnosed with HHT1, and three with HHT2. In the case of the 27-year-old patient, the problem was an unusual bleeding from the oral cavity and throat. This case is an exception due to the origin of the bleeding. Therefore, in this case no ESS was shown, and the patient did not suffer from anemia.

The Epistaxis Severity Score (ESS) was moderate in two cases (around four); however, the reason to be prescribed tacrolimus was anemia due mainly to GI bleeding. In the remaining eight patients, the ESSs were severe. In all cases, the ESSs decreased. Altogether, the ESS decrease was significant, from a mean of 7.3 before tacrolimus treatment to 3 after treatment. This decrease implies a transition from severe to moderate/light epistaxis. Blood transfusions were not required for any of the patients following tacrolimus treatment. This is notable in the cases of P7 and P9, who previously had a need for blood transfusions, as indicated in Table 1. P1, P2, P5, and P6 were receiving iron infusions monthly before treatment. After treatment, their iron infusions were reduced to one every 3 months. The other patients under treatment were not receiving iron infusions (as indicated in Table 1, these patients were on oral iron).

The frequency of office visits did not change in those related to scheduled ones as part of the normal follow-up. However, visits to the office for sclerotherapy were reduced for at least 3 months in 50% of patients (P3, P4, and P10), and no more visits were needed in the other 50% of patients (P8, P9, and P11), since they did not require sclerotherapy. No emergency room visits were recorded since the start of treatment.

In all cases where tacrolimus was well tolerated, the hemoglobin increased significantly. Before tacrolimus, the mean of hemoglobin was 7.9 g/dL. After tacrolimus, the mean hemoglobin value increased to 12 g/dL, very close to normal levels. Of note is the case of P11, where the improvement was astonishing. It is a special case as the patient refused blood transfusions for religious reasons. Before treatments, the patient’s ESS was the highest possible, and the patient’s hemoglobin was extremely low. The sclerotherapy first, and then tacrolimus combined with sclerotherapy, reversed the extremely severe situation of this patient. It is worth mentioning that during treatment with tacrolimus these patients did not have hospital admissions recorded for any type of infection.

**Figure 2:**
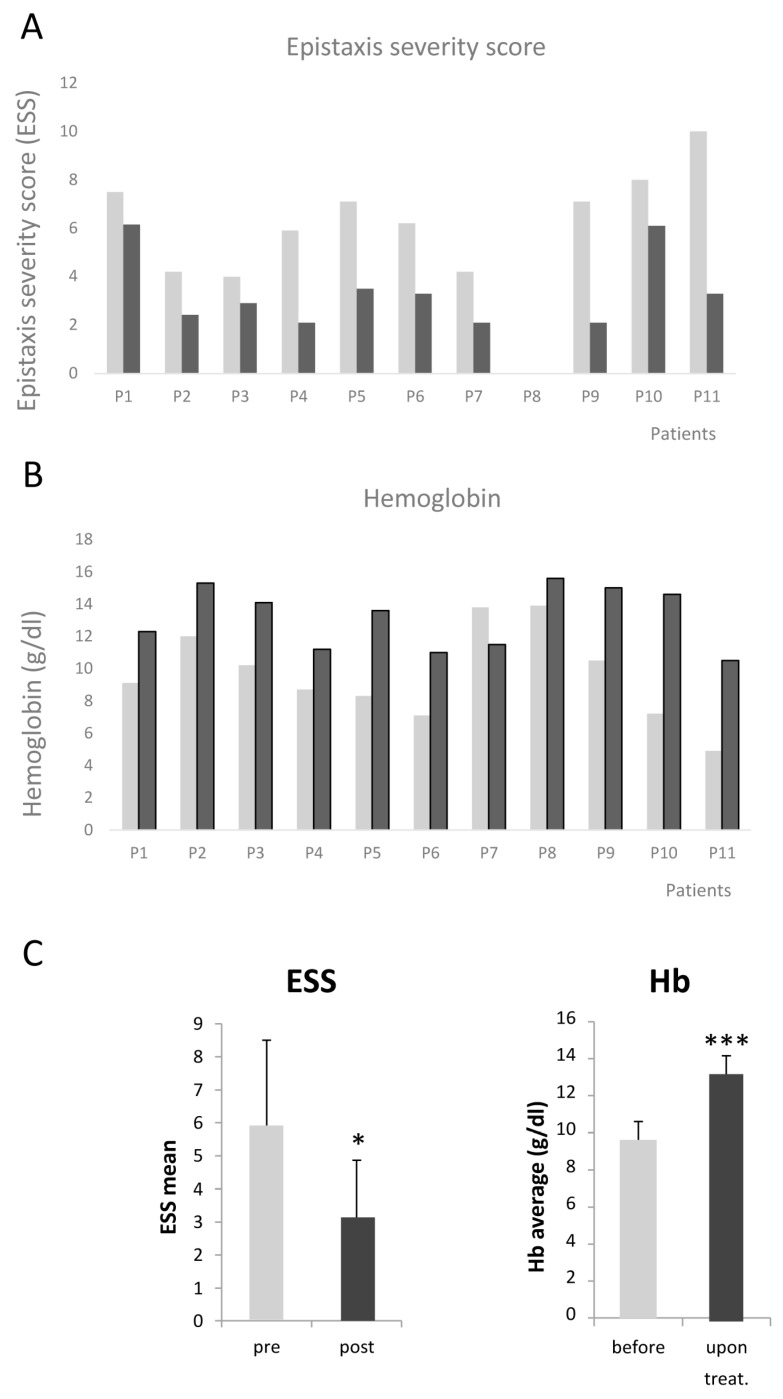
(**A**) Evolution of the Epistaxis severity score in all 11 patients before and after tracrolimus treatment. (**B**) Hemoglobin levels of each patient before and after tacrolimus treatment. (**C**) Mean values of ESS and Hemoglobin levels. * *p* < 0.05, *** *p* < 0.001.

The evolution of ESS and hemoglobin after the initiation of tacrolimus is illustrated in Figure 2.

## 4. Discussion

This study presents the clinical results of 11 HHT patients treated with low doses of oral tacrolimus.

The main objective of this work is to propose tacrolimus as a drug with potential benefits in HHT patients with severe and refractory bleeding (epistaxis or GI), who in some cases are blood transfusion dependent.

The majority of patients treated with tacrolimus showed clear evidence of a substantial reduction in the severity of epistaxis, as indicated by the ESS. On the second hand, these patients also showed an increase in hemoglobin values after the initiation of tacrolimus treatment. As this treatment acts on a systemic basis, the improvement of anemia could be related to a decrease in both nasal and GI tract bleeding.

Nosebleeds represent the most frequently encountered symptom of HHT, and they have a considerable impact on the quality of life of affected patients. Thus, to find promising drugs to control nose bleeds alone, or in combination with other current treatments, is extremely important [25].

To date, there are few pharmaceutical strategies to prevent this complication. Most clinicians recommend the use of local treatments such as moisturizing ointments, oral tranexamic acid, pazopanib, bevacizumab, and,, more recently, the off-label treatment propranolol. However, for very severe bleeding and severe anemia, the management is difficult and depends on the clinician’s expertise and surgical procedures [26].

The benefits of tacrolimus have been noted in isolated cases of HHT patients with liver transplants. However, most of these patients receive immunosuppressive doses of tacrolimus, as indicated, to avoid transplant rejection [19].

Within the clinical context, a study conducted by Sommer and colleagues in 2019 reported a decrease in bleeding in a patient with HHT who also had pulmonary arterial hypertension when treated with low-dose FK506/Advagraf [27]. This case study suggests that a low-dose regimen of tacrolimus (ranging from 0.5 to 1.5 mg/day) is more suitable for patients experiencing resistant nasal or gastrointestinal bleeding, as opposed to the higher doses (5–10 mg/day) typically used for immunosuppression in transplantation [27]. Furthermore, a different report by Hosman et al., which involved two HHT patients reliant on transfusions due to severe bleeding, revealed significant improvement after receiving low-dose tacrolimus treatment [28].

Currently, one active clinical trial for this drug (NCT04646356) is in progress, with the Unit Health of Toronto as the sponsor. This trial is dedicated to examining the efficacy of low-dose oral tacrolimus in addressing recurrent nasal bleeding in individuals with HHT. The primary objective of this trial is to assess the reduction in the severity of epistaxis, measured in terms of the duration of bleeding per week. The study was initiated in 2020, and the anticipated date of completion of the study is September 2024.

Recently, Dupuis Girod et al. [23] published a phase II study with tacrolimus as a nasal ointment showing encouraging results that could lead to a phase III study with a large population.

More recently, Hessels et al. [29] conducted a clinical trial based on 20 patients with HHT who received between 1–2 mg of tacrolimus daily for 20 weeks. The primary end point was the improvement of hemoglobin values. At the end of the study, they found encouraging results; however, 64% of the patients presented minor adverse effects such as headaches, diarrhea, abdominal pain, and insomnia. The patients in this study were closely followed and treated with the standard care during the study. The study participants exhibited quite severe anemia and were of advanced age, potentially hindering efficacy of tacrolimus. Starting patients with tacrolimus at earlier ages, and without such low hemoglobin baseline values (on average 6.1 g/dL, range 5.2–6.9 g/dL), could possibly lead to improved outcomes, as is shown in the present study. It would be worthwhile to explore any potential beneficial effects of administering tacrolimus at varying doses and durations based on patient characteristics (personalized treatments) in a future study.

Some studies show that HHT patients could have an adaptive and innate immunity altered with both a lower lymphocytic count and impaired neutrophil function (NET production, migration, and adhesion). As tacrolimus suppresses immunity by inhibiting T lymphocyte activation and HHT patients could also have a Th1 altered response, the main concern is to follow these patients for potential infectious complications. Further studies would have to be done to evaluate the immunological effect of tacrolimus on HHT patients [30,31,32]. In the present study, we have noticed that lower doses of tacrolimus (0.5 to 2 mg per day) can provide clinical benefits without exposure to higher doses usually indicated for transplant patients. Of note, the adverse effects of this drug are also related to higher doses. As reported in the current study, most of the patients tolerated the medication, with few significant side effects (digestive side effects are the only ones described).

This study is subject to certain limitations due to its cross-sectional design and reliance on retrospective data. Important information, such as modifications in treatment adjustments or detailed records of laboratory analyses, was not gathered. Furthermore, the absence of a protocol outlining inclusion/exclusion criteria is a notable drawback, given the study’s reliance on observational and retrospective data collection. General criteria were primarily based on clinical factors (ESS ≥ 4 and anemia resistant to standard treatments for epistaxis and/or gastrointestinal bleeding). Additionally, expert opinions dictated the dose modifications from the initiation of treatment.

To summarize, this study investigates the potential benefits of low-dose tacrolimus in patients facing severe bleeding and anemia resulting from epistaxis and gastrointestinal losses. Tacrolimus demonstrated efficacy in reducing epistaxis and increasing hemoglobin levels. However, concerns arise regarding potential side effects and the imperative to monitor infectious complications due to altered immunity in HHT patients. Acknowledging its limitations, including the retrospective nature and the absence of an inclusion/exclusion protocol, the study underscores the potential advantages of low-dose tacrolimus in addressing HHT-related bleeding.

It would therefore be important to deepen our knowledge of this drug for HHT in relation to its pharmacodynamics, pharmacokinetics, and potential benefits versus adverse effects.

We can highlight that tacrolimus is a drug with potential use in HHT and that the clinical and molecular results are encouraging. Therefore, it would be interesting to perform clinical trials with more participants to analyze the clinical benefit, quality of life and cost-benefit of tacrolimus in this type of patient.

All together, we may conclude that findings in ongoing trials will hopefully confirm these results in the near future.

## Figures and Tables

**Table 1:** Clinical and laboratory characteristics of hereditary hemorrhagic telangiectasia patients under tacrolimus treatment.

Patient	Dose	Start	End	Tolerance	Sex	Age	Genetics	ESS Pre	ESS Post	Hb before (g/dL)	Hb upon Treatment (g/dL)	Epistaxis, Telangiectasis	Internal Arteriovenous Malformations			Previous Procedures	Previous Drugs
**P1**	1 mg/24 h	2019	-	Good	F	61	Unknown	7.5	6.15	9.1	12.3	Yes	-	HAVMs	CAVM	Embolization right and left external carotid artery branches	Propranolol, raloxifen. IV iron
**P2**	1 mg/24 h	2019	2020	Good	M	61	*ACVRL1*/*ALK-1* c.1208T > C p.L403P Missense	4.2	2.42	12	15.3	Yes	GI AVMs	HAVMs	-	-	Propranolol, bevacizumab IV iron
**P3**	1 mg/24 h	2019	-	Good	F	64	*ENG* c360 + 1G > A	4	2.9	10.2	14.1	Yes	GI AVMs	HAVM (arterio-portal), (Portal Hypertension)	PAVMs. CAVMs. Anemia	Nasal sclerosis Pulmonar embolization	Amchafibrin. Octeotride and oral tamoxifene
**P4**	0.5 mg/24 h	2019	-	Good	F	54	unknown	5.9	2.1	8.7	11.2	Yes	-	HAVMs	Anemia	Nasal sclerosis	Propranolol, amchafibrin. Oral iron
**P5**	0.5 mg/24 h	2019	2021	Digestive Intolerant	M	55	unknown	7.1	3.5	8.3	13.6	Yes	-	HAVMs	PAVMs	-	IV iron
**P6**	1 mg/24 h	2018	-	Good	M	64	Not found in *ENG. ALK1*/*ACVRL1*	6.2	3.3	7.1	11	Yes	-	-	GI AVMs Anemia	Laser for GI AVMs.	IV iron
**P7**	1 mg/12 h	2018	2020	Digestive Intolerant	M	65	*ENG* c.663C > G p.W221X nonsense	4.2	2.1	13.8	11.5	Yes	-	-	-	Inner Maxilar Artery embolization. Young procedure. Sphenopalatine artery embolization.Nasal sclerosis	Amchafibrin Oral iron, blood transfusions, 5 Bevacizumab cycles in 2017
**P8**	1.5 mg/24 h	2018	-	Good	M	27	*ACVRL1*/*ALK1.* Intron 9 c.1377 + 45T > C & c.1377 + 65A > G	0	0	13.9	15.6	Yes	Buccal AVMs	-	-	Nasal Sclerosis	Propranolol
**P9**	0.5 mg/12 h	2018	-	Good	F	51	*ENG*/HHT1	7.1	2.1	10.5	15	Yes	PAVMs	-	Anemia	Nasal Sclerosis	Amchafibrin blood Transfusions
**P10**	0.5 mg/12 h	2021	-	Good	F	47	*ACVRL1*/*ALK1* ex 8 c.1129 G > A p.A377T	8	6.1	7.2	14.6	Yes	-	-	Anemia	Nasal Sclerosis	Oral iron
**P11**	1 mg/12 h	2018	-	Good	M	65	*ENG* ex 5 c.646 A > G p.K216Q	10	3.3	4.9	10.5	Yes	GI AVM	-	Anemia	Nasal Sclerosis	-

ESS: Epistaxis Severity Score; Hb: Hemoglobin; AVM: Arteriovenous malformations; HAVM: Hepatic arteriovenous malformation; PAVM: Pulmonary arteriovenous malformation; F: female; M: male; GI: Gastrointestinal; IV: Intravenous.

## Data Availability

Reported results can be found in the files of the Hospital Universitario Fundación Alcorcón (HUFA) and the Hospital Universitario Ramón y Cajal, Madrid, Spain.
